# Book Reviews

**Published:** 1918-12

**Authors:** 


### BOOK REVIEWS

**English, French, Italian, Medical Vocabulary.** Joseph Marie of Philadelphia, P. Blakiston's Son & Co., Philadelphia. 112 pages, 50 cents.

This is a most useful dictionary with collections of phrases and various useful tables of weights and measures, etc. It is arranged alphabetically according to the English words. At first thought, it might seem that an alphabetic arrangement according to the other languages would be necessary but it is obvious that any one with any familiarity with them would be able to understand a medical expression but would not know just how to word it in translating from English. While appealing especially to surgeons in foreign service, it should be noted that this compend will continue to be of value after the war.

**Manual of Physiology,** G. N. Stewart, M. A., D. Sc., M. D., Published by Wm. Wood & Co., N. Y. 8th edition, 1245 pages, 492 illustrations and colored plates. $5.

We have reviewed earlier editions of this standard text book. It is difficult to comment upon a work so well known and so thoroughly established in professional confidence. Various recent investigations have changed somewhat the point of view especially in regard to internal secretions and ferments. It would perhaps be going too far to claim that radical discoveries have been made since the seventh edition but a clearer grasp of various subjects has been secured.

**Arterio-Venous Aneurysm.** Venot, Jour. de Med. de Bordeaux, Mch., describes a case involving the common carotid and internal jugular, due to a rifle ball (grazing wound implied as no note of haemorrhage) which also caused brachial paralysis. Extirpation, a month later, was successful, the aneurysm having been observed for about 4 weeks.

**Urethral Stricture of Unusual Cause.** Loumeaux, Jour. de Med. de Bordeaux, Mch., observed a case of 10-11 years duration, which had become nearly complete, with dribbling. He operated successfully. The patient had had no venereal disease and the condition was ascribed to repeated contusions in equitation, the man having done a good deal of bareback riding.

